# Anxiety in patients with gastrointestinal cancer undergoing primary surgery

**DOI:** 10.1007/s00432-023-04759-2

**Published:** 2023-04-15

**Authors:** Jens Harms, Benedikt Kunzmann, Jan Bredereke, Lea Harms, Thomas Jungbluth, Tanja Zimmermann

**Affiliations:** 1 Department of Abdominal and Visceral Surgery, Klinikum Wolfsburg, Sauerbruchstrasse 7, 38140 Wolfsburg, Germany; 2 Department of Psychosomatic Medicine and Psychotherapy, Medizinische Hochschule Hannover, Carl-Neuberg-Str. 1, 30625 Hannover, Germany; 3 Department of Gynecology and Obstetrics, Columbia University of New York, New York, USA

**Keywords:** Anxiety, Depression, Gastrointestinal tumor, Surgery, Cancer, Distress

## Abstract

**Purpose:**

Anxiety in the perioperative period is not only an unpleasant emotional state, but can also negatively affect the outcomes and quality of life of surgical patients. The present study investigated anxiety in patients with gastrointestinal cancer scheduled for primary surgery.

**Methods:**

A total of 101 patients in four non-university surgical departments were included. Anxiety (GAD-7), depression (PHQ-9), distress (Distress thermometer), and illness perception (Brief IPQ) were assessed at four time points: first outpatient contact before surgery (t1), preoperative inpatient contact (t2), postoperative inpatient contact before hospital discharge (t3), and postoperative outpatient follow-up contact after 30 days (t4).

**Results:**

56% of patients had an episode of mild or moderate anxiety and 5% had an episode of severe anxiety and/or depression. Subjectively perceived anxiety and depression were highest at t1, followed by t3. 30% of patients had elevated anxiety and depression scores at t1. Regression analyses showed that high subjectively perceived mental distress at t1 was associated with higher anxiety scores at t3 and t4. Women, and younger women in particular, were significantly more likely to experience stress than men. Higher levels of subjectively perceived stress at t1 were associated with higher levels of anxiety at t3 and t4. Sociodemographic factors were not relevant predictors of anxiety.

**Conclusion:**

Anxiety and depression appear to be a persistent problem during the perioperative course in patients with gastrointestinal tumors. Identifying patients at risk for clinically relevant anxiety and depression remains a particular challenge. The results confirm the relevance of repeated screening for mental distress.

## Introduction

Fear remains the oldest and strongest emotion of humanity, and the oldest form of fear is fear of the unknown (Szolloskei 2008). Leading human fears in the healthy population are usually due to dynamic socio-political and economic changes. Fear of illness in the healthy population is not the primary concern.

When hospital treatment becomes necessary in case of illness, everything changes. According to the FORSA survey, people in Germany fear hospital treatment for the following reasons: cancer 65%, treatment errors 65%, treatment failure 61%, pain 53%, risk of death 41% and damage to health due to inadequate care in 41%. Among the greatest fears of illness in Germany is the fear of cancer in 67–73% in the long-term observation by Forsa and Statistica survey (FORSA 2019; Radke 2017). After diseases of the cardiovascular system, cancer diseases represent the second leading cause of death in Germany (Statistisches Bundesamt 2017; Radke 2017). Anxiety and depression are an often hidden but relevant problem and a major source of distress, especially in patients undergoing surgery for tumor diseases. In the general population, elevated levels of anxiety (5.9–7.6%) and depression (15.8–27.5%) are less common than in cancer patients (Hinz et al. 2019; Schwarz et al. 2001). Several studies have shown that between 30 and 40% of oncological patients suffer from elevated anxiety levels (Basak et al. 2015; Caumo et al. 2001; Teunissen et al. 2007). In addition, anxiety and depression scores change during the perioperative course, with anxiety scores decreasing over time (Truong et al. 2019).

Cancer patients undergoing surgery appear to be more affected by anxiety than patients scheduled for chemotherapy or radiation therapy (Truong et al. 2019). Similar findings were observed in patients undergoing cardiac surgery compared with patients undergoing interventional catheter or drug therapy (Truong et al. 2019). Surgery, especially for oncologic patients, can be a drastic life event that not only has a physical impact but also affects the patient’s personal, professional, and economic life. Anxiety in this situation can manifest as fear of the unknown, the unfamiliar place, loss of control over the situation, pain, injury, and fear of complications and especially fear of death (Jawaid et al. 2007).

Although psychosocial symptoms play an important role in the management of oncology patients, these symptoms are often overlooked in the perioperative period of surgical oncology patients. Although these distresses can potentially predict worse patient outcomes, increased postoperative pain, and prolonged hospital stay, as well as increased hospital readmissions after surgery, these distresses should be of real interest to surgical departments and hospital leaders (De Oliveira et al. 2014; Stark and House 2000).

Risk factors for increased anxiety in oncology patients include initial diagnosis, the presence of metastatic disease, dissatisfaction with medical staff and treatment outcomes, inadequate facilities at the treating institution, and higher levels of patient education (Truong et al. 2019). However, demographic variables such as age, gender, marital status, and socioeconomic status are not consistently associated with anxiety in cancer patients (Stark et al. 2002). Active or avoidant coping strategies and social support are helpful in reducing anxiety, while lack of social support and negative illness perceptions can lead to increased anxiety (Broadbent et al. 2006; Karabulutlu et al. 2010; Karakoyun-Celik et al. 2010; Pinar et al. 2012; Zhang et al. 2016).

Most research to date has been conducted with female patients with gynecologic tumors. Therefore, males appear to be underrepresented in clinical studies examining anxiety and related constructs in cancer patients. In addition, it is not clear whether the results in gynecologic tumor patients can be generalized to other tumor collectives in general and to patients with gastrointestinal tumors. Basak et al. analyzed the prediction of anxiety and depression after abdominal surgery in a prospective cohort study and found that at the time of inpatient hospital discharge, 31–56% of the sample had elevated anxiety or depression, respectively (Basak et al. 2015). Depression was predictive of the presence of anxiety in this study. In the same cohort, low educational attainment and low socioeconomic status were the strongest predictors of elevated anxiety (Basak et al. 2015). In another study, risk factors for preoperative anxiety were prior cancer, smoking, mental disorders such as depression, female gender, and an ASA (American Society of Aesthesiologists) category III, which represents patients with severe systemic disease. Furthermore, overestimation of perioperative mortality risk was associated with preoperative anxiety (Caumo et al. 2001; Irlbeck et al. 2017).

Strategies to alleviate anxiety in cancer patients range from patient education (e.g., about postoperative pain control, the disease, and treatment options), psychotherapy, couple- and family-based interventions, art and music therapy, and relaxation techniques (Leitlinienprogramm Onkologie (Deutsche Krebsgesellschaft, Deutsche Krebshilfe, AWMF) 2022; Mirbagher Ajorpaz et al. 2011; Rejeh et al. 2013; Zakerimoghadam et al. 2010). Good communication skills among medical staff appear to improve quality of life and may even lead to better therapeutic outcomes such as shorter hospital stays and lower rates of adverse events (Di Blasi et al. 2001; Harris and Templeton 2001). Similarly, there is evidence that poor communication leads to more anxiety, depression, and poorer quality of life (Lehmann et al. 2009). In addition, fear of recurrence and the impact of coping strategies and perceived disease severity on anxiety in patients undergoing abdominal cancer surgery are still unclear.

To the authors' knowledge, there are currently no comparative studies analyzing the time-dependent dynamics of anxiety during the perioperative period in patients with malignant gastrointestinal tumors undergoing primary surgery. Accordingly, the aim of the present study is to (1) analyze the time-dependent anxiety behavior and level in primary surgical patients with gastrointestinal tumors, (2) identify and verify factors associated with anxiety in the perioperative treatment process, and (3) potentially test the hypothesis whether measuring anxiety and deriving resulting measures can provide an additional quality indicator for the treatment of surgical oncology patients (Lohfert 2013). As described earlier, there are a number of studies analyzing these anxiety-predicting factors, particularly in cardiac surgery and gynecologic tumor patients, but to our knowledge none that have examined the time course and dynamics of anxiety in gastrointestinal tumor patients during the perioperative period in a prospective multicenter study.

## Methods

### Study purpose

The study was a multicenter longitudinal study with four measurement time points. Data for this longitudinal study were collected in four non-university regional large hospitals licensed for tumor therapy in Germany (Lower Saxony, Saxony-Anhalt, and Hesse). Participants were informed about the study in written form and gave written informed consent before participation. Participants completed paper–pencil versions of the questionnaire packet at four defined time points: at the first outpatient preoperative contact in the surgical department (t1), preoperatively at the time of inpatient hospital admission before surgery (t2), at the time of inpatient hospital discharge (t3), and at follow-up 30 days after hospital discharge (t4).

Criteria for study participation included a preoperative histologically confirmed diagnosis of malignant gastrointestinal tumor, primary elective surgery for gastrointestinal tumor according to the appropriate guidelines, age older than 18 years, complete capacity to give informed consent, written compliant consent, and the ability to understand the instructions associated with the study and to answer the questionnaires independently. Acute surgical patients, ASA IV patients, repeat surgery, acute suicidality, and postoperative ICU length of stay greater than six days and death were exclusion criteria for participation in the study.

A positive ethical vote of the Hannover Medical School (MHH) is available (No. 3365–2016). The study protocol was accredited by the German Cancer Society (DKG) for certified cancer centers.

### Measurements

In addition to demographic information, the questionnaire package included validated questionnaires.

#### The German version of the general anxiety disorder questionnaire (GAD-7)

Anxiety symptoms were assessed with the GAD-7 (Spitzer et al. 2006). The items of the GAD-7 correspond to the symptoms of general anxiety disorder in the Diagnostic and Statistical Manual of Mental Disorder (DSM-IV-TR; American Psychiatric Association 2000). Items are rated on a four-point Likert scale ranging from 0 (not at all) to 3 (almost every day). The GAD-7 total score ranges from 0 to 21. Scores of 5, 10 and 15 represent cutpoints for mild, moderate, and severe anxiety, respectively. In this study, internal consistency was good, with Cronbachs α between 0.83 and 0.92 across the four time points.

#### The German version of the patient health questionnaire (PHQ-9)

Symptoms of depression were assessed with the German version of the PHQ-9 (Kroenke et al. 2001). The items of the PHQ-9 correspond to the symptoms of major depressive disorder according to the DSM-IV-TR (American Psychiatric Association 2000). The items are rated on a four-point Likert scale ranging from 0 (not at all) to 3 (almost every day). The PHQ-9 total score ranges from 0 to 27. Scores of 5, 10, 15 and 20 represent cutpoints for mild, moderate, moderately severe and severe depression, respectively. In this study, internal consistency was good with Cronbachs α between 0.79 and 0.89 across all time points.

#### The German version of the NCCN distress thermometer (DT)

The NCCN Distress Thermometer (Mehnert et al. 2006) is a screening instrument for measuring cancer-specific mental distress in oncology patients. It consists of a single item answered on a Likert scale from 0 to 10 and a problem list, which was not used in this study. A cut-off score of ≥ 5 on the thermometer item is recommended and indicates that a patient is under conspicuous stress and needs support.

#### The brief illness perception questionnaire (brief IPQ)

The Brief IPQ (Broadbent et al. 2006) is a nine-item questionnaire that measures a person's cognitive and emotional conceptions of illness. These perceptions are assessed with eight items on a Likert scale ranging from 0 (e.g., “not at all affected”) to 10 (e.g., “extremely affected”). The ninth item is an open-ended item that asks about subjective beliefs about the reasons for the disease and was not used in the present study because only the sum score of the 8 items was used. High scores represent more threatening and negative perceptions of illness. In this study, internal consistency ranged from *α* = 0.73 to 0.76 across the four time points.

### Statistical analysis

The statistical analyses were performed by SPSS® and R (IBM Corp 2021; R Core Team 2018) by an independent investigator blinded to the study. Tests were based if not stated on a significance value of *p* = 0.05. Missing data (28.2%) were handled via multiple imputation using the mice package and the mitml package in R (Buuren and Groothuis-Oudshoorn 2010; Grund et al. 2018). All non-descriptive statistical analyses were performed in a total of 20 multiply imputed datasets before the results were pooled according to Rubin´s rule (Rubin 2004).

With reference to GAD-7 scores group differences anxiety over time were calculated across the four time points using a mixed effects model approach ANOVA via the R package lme4 (Bates et al. 2015). The time points were modeled as a fixed effect and a random effect for each subject was added. The *p* value for the ANOVA was obtained to be a likelihood ratio test of the full model containing the time-point effect against the model without. Turkey´s honest significance test was then used as post-hoc test. *p* values were corrected via Bonferroni correction in the post-hoc analyses (Tukey 1949). Sex differences in GAD-7 scores were tested using t-tests for multiply imputed data included on the MKmisc package (Kohl 2022).

To identify risk factors for increased levels of anxiety at initial contact as well as on the day of hospital discharge, multiple linear regressions were conducted using the GAD-7 score at time points “t1” and “t3” as the respective outcome variables. The predictor variables educational level and relationship status were recoded as dichotomous variables (“low” versus “high”; “in a relationship” versus “not in a relationship”).

To determine the power, an analysis of variance with repeated measures was used for the main objective. To calculate the power, a moderate effect size was assumed. With the help of the program G*Power, the required sample size is *n* = 74 for an alpha error of 0.05 and a power of 80%.

## Results

### Patient sample

A total of 101 patients (*n* = 62 men and *n* = 39 women) were included in the study. Mean age of men was 68.6 years (SD = 11.55; range: 38–86) and did not differ from mean age of women 68.6 years (SD = 10.10; range: 40 – 89; t(99) = 0.017, *p* = 0.986). Demographic characteristics of the patient sample are summarized in Table 1.

**Table 1:** Demographic and cancer-related characteristics of the sample (*n* = 101)

	Men	Women	Total	*P*^c^
	(*n* = 62)	(*n* = 39)	(*n* = 101)	
Type of Cancer (*n*, %^a^)				0.090^b^
Esophagus	3 (5%)	–	3 (3.06%)	
Stomach	1 (1.67%)	3 (7.89%)	4 (4.08%)	
Pancreas	2 (3.33%)	4 (10.53%)	6 (6.12%)	
Colon and rectum	54 (90%)	31 (81.58%)	85 (86.73%)	
UICC stadium (*n*, %^a^)				0.381^b^
0	1 (1.64%)	–	1 (1.02%)	
1	26 (42.62%)	19 (51.35%)	45 (45.92%)	
2	17 (27.87%)	8 (21.62%)	25 (25.51%)	
3	10 (16.39%)	9 (24.32%)	19 (19.39%)	
4	7 (11.48%)	1 (2.7%)	8 (8.16%)	
Age (M, SD)				0.986
	68.63 (11.55)	68.59 (10.10)	68.61 (10.96)	
Education (*n*, %^a^)				0.576^b^
< = 9 years of school	19 (52.78%)	10 (35.71%)	29 (45.31%)	
10 years of school	10 (27.78%)	11 (39.29%)	21(32.81%)	
> 10 years of school	3 (8.33%)	3 (10.71%)	6 (9.38%)	
Other	4 (11.11%)	4 (14.29%)	8 (12.5%)	
Relationship status (*n*, %^a^)				0.090^b^
Single	2 (5.56%)	–	2 (3.08%)	
Married/in a relationship	23 (63.89%)	15 (51.72%)	38 (58.41%)	
Divorced	7 (19.44%)	4 (13.79%)	11 (16.92%)	
Widowed	4 (11.11%)	10 (34.48%)	14 (21.54%)	

^a^All percentages were calculated using valid cases only and sums of more than 100% are due to the rounding to two decimal places

^b^Fisher’s test

^c^Of the sex comparison

The majority of the patients (58.4%) lived in a relationship. In terms of educational status, more than half of the patients had up to 10 years of schooling. There was no gender difference in terms of education (*χ*^2^ (3) = 1.87, *p* = 0.60).

Colorectal cancer (*n* = 85; 86.73%) was the most common oncologic diagnosis, along with pancreatic cancer (*n* = 6, 6.12%), gastric cancer (*n* = 4; 4.08%) and esophageal cancer (*n* = 3; 3.06%). Regarding the postoperative pathological tumor classification based on the International Union Against Cancer (UICC) staging system, most of the resected specimens had localized tumor stages (UICC I) in 45.92%, followed by locally advanced tumor stages (UICC II and III) in 44.9% of cases. Patients with distant metastases corresponding to a UICC IV stage formed a minority with 8.16%.

### Anxiety

Overall, *n* = 56 patients (55.45%) experienced anxiety symptoms at least once during the perioperative treatment course that could be classified as mild or moderate according to their respective GAD-7 scores. In *n* = 5 (4.95%) participants, symptoms above the threshold for severe anxiety and/or depression occurred during the treatment phases. Of the *n* = 56 patients with mild/medium anxiety, *n* = 17 patients (33%) experienced mild to moderate anxiety at time t1 (first outpatient prehospital contact).

### Anxiety over time

Anxiety was most prevalent immediately at the first outpatient contact (t1) and before postoperative inpatient hospital discharge (t3). At the preoperative inpatient time point (t2), anxiety scores were lower than at t1 and t3. The lowest anxiety scores were observed 30 days after hospital discharge at the outpatient follow-up (t4).

**Fig. 1:**
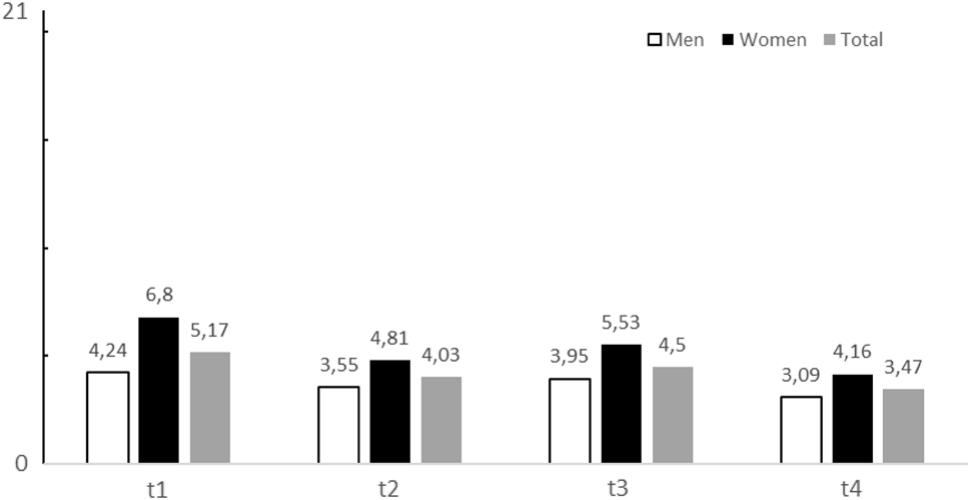
Anxiety mean scores (measured with GAD-7) at different time points (t1 = ambulant prehospital outpatient contact, t2 = preoperative inpatient hospital contact, t3 = postoperative inpatient contact prior hospital discharge, t4 = postoperative 30 days outpatient follow-up contact) of the total sample (*n* = 101, *n* = 62 men, *n* = 39 women)

A likelihood ratio test that compared a model for GAD-7 scores with the factor time point with a reduced model that did not include the factor indicated a significant effect of the time point (3558.79) = 3.675, *p* = 0.01; see Fig. 1). Post-hoc tests were then utilized to identify which time point differed from each other. This post-hoc analysis initially suggested that t1 and t2 (*p* = 0.017), as well as t1 and t4 (*p* = 0.001) differed significantly while the pairwise comparisons of the t1 and t3 (*p* = 0.141), t2 and t3 (*p* = 0.424), t2 and t4 (*p* = 0.439), as well as t3 and t4 (*p* = 0.099) were not statistically significant. To compensate for this second evaluation of the factor time point, a Bonferroni correction was used. After correction only the difference between t1 and t4 remained statistically significant (corrected *α* = 0.008).

### Risk factors for increased anxiety

A first multiple linear regression model (pooled *R*^2^ = 0.61) was calculated to predict patients GAD-7 scores at t1 based on the following variables: sex, age, relationship status, educational level, distress at t1, and depressive symptoms at t1. Moreover, patients GAD-7 scores at t3 were predicted via second multiple linear regression model (pooled *R*^2^ = 0.42) including the following additional variables as predictors: anxiety scores at t1 and BIPQ scores at t2 (see Table 2).

**Table 2:** Results of the multiple regression analyses (*n* = 101 patients)

Model	*B*	SE	*t*	*p*
Anxiety (t_1_)				
Intercept	5.58	2.53	2.21	0.032*
Sex	0.56			0.29
Age	− 0.06			0.07
Relationship status	0.26			0.61
Educational level	0.66			0.25
Distress thermometer (t_1_)	0.30			0.001*
PHQ-9 (t_1_)	0.50			0.001*
Anxiety (t_3_)				
Intercept	− 1.12			0.69
Sex	0.57			0.30
Age	0.01			0.82
Relationship status	0.22			0.66
Educational level	0.23			0.68
Distress thermometer (t_1_)	0.10			0.50
PHQ-9 (t_1_)	0.04			0.71
GAD-7 (t_1_)	0.27			0.12
Brief IPQ (t_2_)	0.08			0.02*

PHQ-9 (Patient Health Questionnaire), GAD-7 (General anxiety disorder questionnaire), brief IPQ (brief illness perception questionnaire)

At t1, factors for anxiety were depression (*p* = 0.001) and psychological distress (*p* = 0.001). Anxiety, depression and psychological distress at t1 were otherwise not predictive for anxiety at t3. Only illness perception at t2 showed a small correlation with anxiety at t3. This result indicates that a prominent screening with the distress thermometer has no predictive value for the occurrence of anxiety symptoms at a later time point.

The influence of sex, marital status and educational status were not statistically significant in the multiple regression analysis. However, there was a trend for higher anxiety levels on younger patients. Using t-tests, women over all different time points showed higher levels of anxiety than men. There were statistically differences between the sexes regarding levels of anxiety at the t1 (*t* = − 2.686, df = 54.895, *p* = 0.01) with women showing higher levels of anxiety then men. At t2 (*t* = − 1.8058, df = 60.199, *p* = 0.076), t3 (*t* = − 1.724, df = 39.937, *p* = 0.092), and t4 (*t* = − 1.087, df = 43.934, *p* = 0.283) no statistically significant differences between the sexes were observed. These observations were in line with the theory that anxiety over time behaves dynamically in the perioperative period. Comparative boxplot analysis of sex, age, and measures of distress at t1 showed that, compared with men, women with ages younger than 50 years were more likely to have elevated levels of anxiety. This observation could not be reproduced for men (see Fig. 1).

## Discussion

In general, the literature suggests that patients undergoing surgery for cancer are more likely to be anxious than patients not undergoing surgery or patients undergoing surgery for a benign, noncancerous condition. Surgical procedures in cancer patients involve more existential issues than in other surgical patients. Anxiety in surgical patients generally includes fears of pain, alienation, loss of autonomy, sometimes acute loss of perspective on life, and, at least in oncologic patients, fears of death. In addition, treatment outcome and response to tumor therapy have a major impact on patient anxiety. Anxiety and depression in general can have a negative impact on surgical patients, leading to dysfunction, longer hospital stay, less effective treatment outcomes, and increased rates of hospital readmissions (Stark and House 2000).

In the present study, elevated anxiety was found in 56% of patients. This frequency is consistent with the findings of Basak et al. (2015), who found a frequency of anxiety ranging from 31 to 56% in patients undergoing abdominal surgery at the time of hospital discharge. Over time, anxiety decreased among patients in this study. This is consistent with Hinz et al. (2009) who showed that patients were more anxious at baseline and had higher psychological stability later. Since anxiety in the perioperative period is not a static phenomenon, it can be assumed that the content of the underlying psychological discomfort is also subject to dynamic changes. Moreover, in the present study, the level of anxiety behaved inversely proportional, especially between t1 and t4, with a maximum at t1 and a minimum at t4. Regarding the time points recorded during the perioperative period, anxiety seems to be relevant at the time of the first prehospital outpatient contact.

This finding is consistent with studies that found that anxiety often occurs when the disease is first diagnosed or communicated, when the decision about primary surgical treatment is made based on the results of tumor staging, or when they feel anxious about the extent of tumor disease (Delibegovic and Sinanovic 2013; Holland 1989). The observation of the maximum anxiety level at t1 and t3 was also striking. Both time points were associated with a change in treatment sector from outpatient to inpatient and vice versa. This finding illustrates that for surgical tumor patients, the change of treatment sector may represent the greatest emotional burden with presumably varying negative content.

In this context, the question also arises as to the supposedly different causes of manifested anxiety at different times in the perioperative phase. The influencing factors for a most frequent manifestation of fears at the time of the first appointment in the surgical department (t1) can be, as sometimes observed, in the lack of information about the underlying diagnosis, in information deficits regarding the extent and the general prognosis of the tumor disease, in the unclear acute more or less dramatic loss of life perspective, in the unfamiliar hospital environment, in existential fears, in the fear of loss of autonomy and self-determination in the clinical setting with regard to surgery and anesthesia, and not least in the fear of death. At this point, it is also important to consider the aspect that in a priori stressed patients, the feeling of anxiety at this time may be exacerbated in the absence of preventive psycho-oncological support. All these aspects underline the urgent need for ultra-short patient distress screening, possibly using the distress thermometer already at the first prehospital outpatient contact.

Patients' preoperative anxiety levels were lower at t2 than at t1 and t3, but higher than at t4. At t2, anxiety was most likely due to the upcoming surgery in combination with anxiety about anesthesia and surgery and possible complications. Other possible factors include fear of loss of autonomy and control, fear of pain, fear of not waking up after the surgical procedure, and fear of death. However, some reduction in anxiety must be considered at this time, which may be due to assimilation or explicit adaptation to the given circumstances, or an expression of anxiety that reduces competent treatment by medical and nursing staff.

Anxiety scores before inpatient hospital discharge (t3) were similar to the second anxiety score in the perioperative period. Anxiety scores did not differ significantly and did not exceed the first maximum anxiety score at the first prehospital outpatient contact (t1). Otherwise, anxiety scores were higher compared with the inpatient prehospital time point (t2) and moderate compared with the outpatient time point of 30 day follow-up after hospital discharge (t4). Factors explaining this second spike in anxiety at time t3 could be related to the lack of a clear diagnosis of the pathologic specimen, lack of disease management, uncertainty about the overall oncologic prognosis, wound healing not yet completed and surgical sutures not yet removed, persistence of unregulated bowel function, and fear of needing help from others in the outpatient setting.

At t4, the 30 day outpatient follow-up after hospital discharge, anxiety scores were not significantly lower than at the previous t3 immediately before hospital discharge. Anxiety scores at the outpatient follow-up at t4 otherwise decreased significantly compared with anxiety scores at the first outpatient contact before hospitalization (t1). The persistence of anxiety or the decrease in anxiety at t4 may be due to successful or pending reintegration into out-of-hospital social structures, pending or complete reintegration into working life, but also to coping optimism or optimism of purpose in the case of waiting for adjuvant chemotherapy based on the results of pathologic examination of the resected specimen.

In conclusion, without considering the number of unreported cases, more than half of the patients experienced anxiety at least once during the perioperative course. It must be noted that anxiety levels do not behave statically during the perioperative course in patients with gastrointestinal tumors in whom primary surgery is planned. In general, anxiety behaves dynamically during the perioperative course and is therefore less predictable. The combination of anxiety and depression was predictive of persistent disturbance during the perioperative course. Anxiety appeared to be particularly more pronounced at the times when the patient’s treatment areas were about to change. In the current study, this corresponds first to the time of initial prehospital outpatient presentation to a surgical department (t1) and second to the time postoperatively immediately before inpatient discharge to the primary care outpatient setting (t3).

Other studies have found that several factors are associated with anxiety in cancer patients, including sociodemographic factors, functional status, and social support (Gonzalez-Saenz de Tejada et al. 2017; Kim et al. 2017; Koivula et al. 2002; Truong et al. 2019). These studies primarily address the spectrum of oncological collectives in general and patients after cardiac surgery from Asian or Northern European populations. In the current study, some disease-related factors but not the entire spectrum were analyzed. For example, the impact of postoperative tumor extent based on the pathologic UICC classification was not analyzed because of the heterogeneity of the included gastrointestinal tumor entities, incongruence of postoperative tumor stages (e.g., UICC 0 and UICC IV versus UICC I, II, III), and pathologic investigator-dependent bias in a multicenter study. The number of patients with advanced metastatic tumor disease (UICC IV) formed a minority in the patient sample. In addition, patients with advanced tumor disease often have higher ASA classification scores and are more likely to require a multidisciplinary therapeutic approach and no surgery initially, and are at higher risk of dying during the perioperative course, so they were excluded from the study.

Sociodemographic factors such as partnership and education level, except for a statistically significant difference in gender with predominant manifestation of anxiety in female patients, were not related to the likelihood of anxiety disorder. In general, females exhibited more frequent and more pronounced anxiety compared to males across all time points. Again, these differences were most apparent at the overlap of care settings, ie, at time points t1 and t3. The reason for the gender difference can generally be assumed to be that men have different perceptions and different strategies for coping with cancer and surgery than women. In addition, the predominant willingness to manifest anxiety in women may be due to their social role in the family network, partnership, and also as a divorced or widowed single person.

Women younger than 50 years more often showed salient anxiety scores at time t1. This may be due to age-related differences in their role in the family network, life planning not yet completed, cosmetic aspects and limitations after surgery, and fear of dying younger. Older female patients are likely to have longer and greater life experience, have completed their family planning, may have a history of disease, and are less involved with obligations in the family social network. Without neglecting men, two consequences should be drawn from these observations: (1) Screening for mental distress should focus particularly on the group of women and specifically on the subgroup of young women (< 50 years of age). (2) Screening should be repeated especially at the intersectoral boundaries between the outpatient and inpatient sectors, because this is where anxiety manifestations were most frequent and severe in both sexes.

## Limitations

Limitations to the power of the study resulted from the unbalanced gender distribution, main oncologic diagnoses, and varying recruitment rates per participating hospital, variable site-specific factors, and demographic influences of the participating hospitals. In this study, patients with advanced cancer, i.e., metastatic disease (UICC IV), represented a minority of 8.16%. Consequently, no conclusion can be drawn in an intergroup validation as to whether patients with advanced tumor stage, are associated with a higher anxiety burden than patients in lower tumor stages. The results cannot be related to all gastrointestinal cancers because, for example, tumors of the esophagus, stomach, liver, and pancreas were underrepresented. Treatment of these tumors is often multimodal, with surgery often forming the second line, being more complex and associated with a higher risk of postoperative morbidity and death. Further research is needed to investigate anxiety in these patients. Colorectal carcinomas represent the majority of cancer diagnoses in this study, accounting for 86% as one of the most common gastrointestinal tumors in the Western world. Colorectal carcinomas are generally diagnosed through screening programs, resulting in most cases being diagnosed at a localized, non-advanced stage. In most cases, primary surgery is the treatment of choice after completion of staging. However, the results of our study are primarily representative of colorectal cancer patients.

In addition, given the possible denial and denial of discomfort and anxiety at the time of completion of the study questionnaire, it must be assumed that there is an estimated underreporting of discomfort and anxiety, so the incidence and magnitude of anxiety may be even higher than the present data suggest.

A study by Sheppard et al. (2014) found that patients who had high opinions of health care professionals’ professional competence, interpersonal skills, and hospital facilities had lower levels of anxiety. It is likely that this effect could play out at t2, t3, and t4 in the current study. Because this study followed a multicenter approach with 4 different hospitals in different urban localizations, a general and specific assessment of this effect was statistically revealed. In this study, an effect on this item could be expected at time t2, when, by nature, a further spike in anxiety levels would be expected immediately before the upcoming surgery. In contrast, a decrease was observed that was below the anxiety level at t3. The results support the proposition that positive communication with patients, a patient-centered approach to care, and an appropriate environment per se improve the relationship/interaction between patients and health care professionals, thereby reducing distrust of health care professionals and service quality, and ultimately alleviating patient anxiety (Troung et al. 2019).

## Conclusion

Study findings suggest that, as with other oncologic conditions, anxiety is a burden in patients with gastrointestinal cancers for whom primary surgery is the treatment of choice. More than half of the study population had at least mild anxiety throughout the perioperative period, and 5% had moderate or severe anxiety. It is likely that the prevalence of hidden anxiety is even higher than that recorded in this study. The magnitude and course of anxiety during the perioperative course are not predictable prima vista after baseline assessment, with the exception of those patients who already showed abnormal values at screening with the distress thermometer before hospital admission.

Anxiety in the perioperative course behaves dynamically. Therefore, repeated psycho-oncological screenings should be introduced and become standard during the perioperative course to respond appropriately to the patient's psychological state. If complex neoadjuvant therapy concepts become necessary, such as in advanced tumor stages, it is also advisable in view of the study results to integrate repeated sequential distress screenings throughout the treatment period. The same applies to the recently introduced fast-track and prehabilitation concepts. Fast-track concepts aim to keep the duration of inpatient hospitalization as short as possible and necessary. Prehabilitation concepts, on the other hand, aim to preoperatively improve the outcome chances of patients in high-risk constellations, with psychooncology as one of the main pillars along with nutritional counseling, physiotherapy, and drug treatment (Frank et al. 2022).

Anxiety in patients with gastrointestinal cancer seems to be particularly pronounced and frequent when a change in treatment is imminent and vice versa. This corresponds first to the time of initial prehospital outpatient presentation in a surgical department and second to the time postoperatively immediately before inpatient discharge to the primary care outpatient setting. These two time points, among others, are key points for psycho-oncological screening, e.g., with the Distress Thermometer. If abnormal values are detected, at least psycho-oncological counseling should be offered.

In addition, some practical consequences must be drawn from the study results. Women, especially younger women, seem to be more frequently and intensively affected by anxiety disorders, so that screening should especially focus on this subgroup of women. On the other hand, screening in men should not be neglected and, if necessary, ways should be sought to decipher men’s specific coping strategies. This also raises the question of how anxiety behaves in young men, since the age distribution in the present study sample ranged from 38 to 86 years.

At the initial prehospital outpatient presentation with oncologic disease, the diagnosis should, in the best case, already be known to the patient. This facilitates communication about the disease and surgical therapy. The task of educating the patient about the diagnosis is ideally the responsibility of the referring primary care physician. Accompanying family members to this initial consultation may reduce the patient's anxiety. Regardless, initial psycho-oncology screening should occur at this initial contact. If there are any abnormalities, the surgeon should already recommend the offer of psycho-oncological counseling as a preventive measure.

To avoid further uncertainties, it should be possible to inform the postoperative patient about the result of the pathological examination of the resected tumor specimen and any further therapy that may be necessary, at least in the form of adjuvant radio- or chemotherapy, before transfer to the out-of-hospital setting at home. This could be another quality feature for surgical tumor treatment in the future.

## Data Availability

The datasets generated during and/or analyzed during the current study are available from the corresponding author on reasonable request.
